# Impaired pitch identification as a potential marker for depression

**DOI:** 10.1186/1471-244X-12-32

**Published:** 2012-04-19

**Authors:** Michael Schwenzer, Eva Zattarin, Michael Grözinger, Klaus Mathiak

**Affiliations:** 1 Department of Psychiatry, Psychotherapy and Psychosomatic Medicine, RWTH Aachen University, Pauwelsstr. 30, D-52074 Aachen, Germany

**Keywords:** Depression, Non-verbal processing, Pitch identification, Motivation, Sex effect

## Abstract

**Background:**

Impaired auditory performance has been considered as marker for depression. The present study tested whether pitch perception is affected in depression and whether the impairment is task-specific or reflects global dysfunction.

**Methods:**

Twelve depressive in-patients and 12 non-depressive participants, half of the sample women, volunteered. The participants performed pitch identification using a four-choice reaction task, pitch contour perception, and pitch discrimination.

**Results:**

During pitch identification but not during pitch contour perception or pitch discrimination, depressive patients responded less accurate than non-depressive participants (*F* = 3.3, *p* = 0.047). An analysis of covariates revealed that only female but not male depressive patients identified pitches poorly (*Z* = −2.2, *p* = 0.025) and inaccurate pitch identification correlated with high scores in the Beck Depression Inventory in women (*r* = −0.8, *p* = 0.001) but not in men (*r* = −0.1, *p* = 0.745). Patients did not differ from controls in reaction time or responsiveness.

**Conclusions:**

Impaired pitch perception in depression is task-specific. Therefore, cognitive deficits in depression are circumscribed and not global. Reduced pitch identification in depression was associated with female sex. We suggest that impaired pitch identification merits attention as a potential marker for depression in women.

## Background

Cognitive deficits in depression arise among others in the auditory domain [1]. Thus, some authors considered impaired auditory processing as a potential marker for depression [2,3]. There is a controversy, however, whether cognitive dysfunctions in depression are specific [4] or general [5,6]. Variable findings of correlations between depression and pitch perception may indicate the involvement of specific impairments: depressive patients responded less [1], similarly [7], or even more accurately [8] than healthy controls in pitch perception tasks. The variable outcomes suggest that heterogeneous processes may be differentially affected during a depressive episode. The present study aimed to confirm that a specific pitch perception skill - pitch identification - and not pitch perception in general is impaired in depressive patients.

Three different skills contribute to pitch perception: pitch identification, pitch contour perception, and pitch discrimination [9,10]. There are reasons to hypothesize that depression impairs specifically pitch identification: Pitch identification was assessed using a choice reaction task [11], and visual choice reaction was poor in depression [6,12,13]. Moreover, pitch identification activated the intraparietal sulcus which was associated with numerosity encoding [11], and non-verbal encoding strategies were impaired in depressive patients [14]. In contrast, little data support that depression affects pitch contour perception or pitch discrimination.

The test design can bias outcomes in a clinical study. For instance, too easily distinguishable pitches could level out differences due to a ceiling effect. A ceiling effect may explain why Knott et al. found no impairment of auditory perception in depression [7]. Indeed, depressive patients performed poorly only during effortful tasks [15]. A second reason for variable outcomes may be a speed-accuracy tradeoff in depressive patients as described by Kalb et al. [8]. Thirdly, reduced performance may reflect low motivation to respond, i.e. poor ‘responsiveness’ [1,16]. To minimize misinterpretations due to such confounding factors, auditory tests in depression should employ difficult tasks and monitor speed-accuracy tradeoff and responsiveness.

The present investigation applied pitch identification, pitch contour perception, and pitch discrimination tasks which reflected different auditory skills and neurophysiologic processes [10,11,17]. The tests were adjusted to high task difficulty by selection of small pitch variations such that the tests were sensitive to impairments [10,15]. To detect a potential speed-accuracy tradeoff, reaction times were recorded. Moreover, we considered the sum of correct and incorrect responses as measure of responsiveness.

Based on the quoted literature the hypothesis was tested, that auditory accuracy during pitch identification but not during pitch contour perception and pitch discrimination is impaired in depressive patients. This finding was expected to emerge independent of potential covariates, i.e. speed-accuracy tradeoff, responsiveness, motivation as well as sex, age, musical experience, education, and smoking.

## Methods

### Participants

Twelve patients suffering from unipolar depression and 12 healthy controls volunteered. Half of each sample was women. The depressive participants were in-patients of the RWTH Aachen hospital. Depression was diagnosed according to ICD-10 [18] by at least two experienced psychiatrists including MG. Table 1 displays the diagnosis for each patient. None of the patients had hallucinations. Eleven patients were medicated (5 x mirtazapine, 3 x venlafaxine, and 3 x atypical antipsychotics) for at least four weeks; one patient was not medicated. The healthy participants had no mental disorder according to a standardized clinical interview [19] and were free of medication. Both groups comprised three musicians and nine non-musicians each. All participants were right-handed as determined by scores above 67 in the Edinburgh Handedness Inventory [20]. All participants were naïve to the auditory tasks and had not taken part in a similar study before. The participants reported not to be hard of hearing. In the practicing trials of each auditory test, the participants were asked whether they could hear each tone, which was the case. All participants volunteered on the base of written informed consent and were paid. The medical ethics committee of the Aachen University approved the study.

**Table 1:** Characteristics of depressive patients and non-depressive controls

**ICD-Diagnosis**	**BDI**^a^	**sex**	**age**	**musician**	**education**^**b**^	**smoker**
Depressive patients						
F32.1	19	f	36	no	low	yes
F32.1	23	f	47	no	low	yes
F32.2	23	f	62	yes	high	no
F33.1	27	f	48	yes	high	yes
F33.1	27	f	63	no	middle	no
F33.2	29	f	55	no	low	yes
F32.1	15	m	24	yes	high	no
F32.2	31	m	27	no	high	yes
F33.2	34	m	36	no	high	yes
F33.3	11	m	52	no	high	yes
F33.3	34	m	40	no	low	yes
F33.3	17	m	55	no	high	no
M ± SD	24 ± 7	6/6	45 ± 12	3/9	4/1/7	8/4
Controls						
	4	f	47	no	middle	yes
	8	f	51	no	middle	yes
	8	f	60	yes	middle	no
	1	f	50	yes	high	yes
	0	f	62	no	middle	no
	1	f	58	no	low	yes
	3	m	22	yes	high	no
	5	m	23	no	high	yes
	1	m	39	no	high	no
	2	m	56	no	middle	yes
	0	m	42	no	middle	yes
	1	m	52	no	high	yes
M ± SD	3 ± 3	6/6	46 ± 13	3/9	1/6/5	8/4

^a^ BDI = scores in the Beck Depression Inventory.

^b^ low = no secondary modern school; middle = secondary modern school education; high = matriculation standard.

### Measurements

#### Auditory performance

A personal computer equipped with a custom program presented the pitch identification, pitch contour perception, and pitch discrimination tasks [10]. The computer soundcard produced sine wave tones (100 ms with 10 ms onset/offset ramps, 52 dB). The tones were played after a silent period varying randomly between 100 and 900 ms. In each task, different types of trials occurred in pseudo-randomized order with the constraint that the type changed between consecutive trials. The participants listened to the tones via headphones that absorbed ambient noise and responded on a 4-button keypad. The program recorded each response within 1.8 s after stimulation. Each trial lasted 3 s regardless of the variable silent period and the response time. The program registered each response and the reaction time of correct responses.

During the *pitch identification* task, one of four frequencies (800, 832, 852, and 872 Hz) were played in each of 60 trials. The frequencies did not represent a standardized, e.g. chromatic, linear or logarithmic scale, to avoid a bias due to the recognition of regularities. The participants memorized each frequency before the measurement and were instructed to identify the tones independent from preceding trials. The participants categorized the pitches on the 4-button keypad. The keys indicated rising frequencies from left to right, i.e. pressing the index finger after 800 Hz, pressing the middle finger after 832 Hz etc.

During *pitch contour perception*, the participants should detect a pitch ascent within a melody of descending pitches. In 60 out of 120 trials (resp. melodies), a pitch ascent occurred. Each melody consisted of a dichotic sequence of four tones in which the sequence of pitches differed between ears. For instance, pitches were 683, 724, 645, 215 Hz at the left ear, and 1149, 1149, 966, 304 Hz at the right ear listed in temporal succession; in this example the participant should notice the pitch ascent from 683 to 724 Hz at the left ear. Four basic melodies were generated; left and right side were balanced additionally and varied with respect to pitch ascent vs. no pitch ascent. Considering all melodies, the tonal range was between 304 and 3068 Hz, the pitch ascended up to three semitones, the descents of pitches were up to 20 semitones between subsequent tones. We chose such a complex presentation to achieve a high task difficulty and pitch contour perception should clearly differ from the pitch identification and pitch discrimination tasks. The participants had to press a fixed button of the 4-button keypad whenever the pitch rose; unused keys were covered to reduce distraction. The participants were instructed to avoid false positive responses.

In the *pitch discrimination* task, two succeeding tones had the same frequency or the second tone was higher than the first tone, i.e. an ascent from 1000 to 1004, 1008, 1012, or 1016 Hz. The frequency ascended in 60 out of 120 trials whereas in the other half of trials the frequency remained 1000 Hz in both succeeding tones. Like during pitch contour perception, participants should press the fixed button of the 4-button keypad when the frequency increased and avoid false positive responses.

#### Assessment of personal data

All participants completed the *Beck Depression Inventory* (BDI [21]; German version [22]). For assessing *self-rated motivation*, the participants stated on a scale from 1 through 10, how much they made an effort to achieve a good performance in the testing. For the assessment of *musical experience*, the investigator inquired how often the participant played an instrument or sang in a choir, how many years he played the instrument or sang, and if he took lessons. Since all German pupils received music lessons in school, only practice or lessons in addition to regular school education was considered. Playing an instrument or singing in a choir at least once per week during a period of two years distinguished musicians from non-musicians in this study.

### Procedure

After informed consent and the clinical interviews, the participants completed the BDI and the Edinburgh Handedness Inventory. Pitch identification, pitch contour perception, and pitch discrimination were accomplished in randomized order. The investigator introduced each pitch perception test, played each kind of trial, and demonstrated the expected responses. In each task, the participants performed 60 practice trials before assessment. After the auditory assessment, the investigator enquired self-rated motivation.

### Statistics

The analysis considered the number of correct responses as measure of accuracy. A correct response meant pressing the correct key when a response was expected. Pressing a false key when a response was expected or pressing any key when no response was expected were valued incorrect responses. Trials without pressing a key were ignored. The average reaction time of correct responses allowed analyzing the speed-accuracy tradeoff. The sum of correct and incorrect responses should indicate responsiveness and may be an indicator of drive together with the motivation rating.

The Shapiro-Wilk test assessed Gaussian normal distribution of data as a prerequisite for parametric analysis. An ANOVA tested the hypothesis that pitch perception is impaired in depressive compared to non-depressive participants. The interaction between the group factor (depressives vs. controls) and the repeated measurement factor of pitch perception (pitch identification, pitch contour perception, pitch discrimination) as well as post-hoc *t*-tests should confirm a task-specific effect. Not normally distributed data and covariates with cells with six or fewer participants were analyzed using the non-parametric Mann–Whitney U-test or a *χ²*-test for more than two categories. Pearson’s correlation coefficients and regression analysis allowed further data exploration. The significance level was *p* < 0.050. A Bonferroni correction was applied.

## Results

Most data approximated a normal distribution (*S-W* > 0.93, *p* > 0.110) but the sum of correct and false responses in pitch identification (*S-W* = 0.57, *p* = 0.001) and self-rated motivation scores (*S-W* = 0.90, *p* = 0.025) yielded a ceiling effect. A task-specific impairment emerged (interaction depression x tasks: *F*(2,44) = 3.3, *G-G* = 0.98, *η²* = 0.13, *p* = 0.047). Post-hoc tests revealed that depressive patients identified pitch less accurately than controls without impairment in pitch contour perception and pitch discrimination (Table 2); during pitch identification, depressive patients yielded 24% fewer correct responses than non-depressive participants. Depression did not slow-down response times (interaction depression x tasks: *F*(2,44) = 0.4, *G-G* = 0.96, *p* = 0.620). No significant speed-accuracy tradeoff emerged during pitch identification (*r*(24) = −0.19, *p* = 0.368) and pitch contour perception (*r*(24) = −0.22, *p* = 0.297) but during pitch discrimination (*r*(24) = −0.47, *p* = 0.020). Responsiveness did not differ between depressive and non-depressive participants (interaction groups x tasks *F*(2,22) = 0.5, *G-G* = 0.96, *p* = 0.575; main difference between groups *F*(1,11) = 0.1, *p* = 0.793). Thus, responsiveness cannot explain differential accuracy scores. Self-rated motivation was identical in depressive and non-depressive participants (*Z* = −0.1, *p* = 0.910).

**Table 2:** Pitch perception and motivation in depressive patients and non-depressive controls

	**Depressives N = 12**	**Controls N = 12**	**Difference**	**t/Z**^a^	**p**
	**M ± SD**	**M ± SD**	**M ± SD**		
*Accuracy*					
Pitch identification	23.7 ± 10.6	31.4 ± 6.0	−7.6 ± 12.0	−2.1	0.040*
Pitch contour percept.	27.5 ± 10.1	23.9 ± 11.4	3.5 ± 17.3	0.8	0.426
Pitch discrimination	26.6 ± 13.7	25.3 ± 11.5	1.3 ± 18.2	0.2	0.799
*Reaction time (ms)*					
Pitch identification	1013 ± 234	954 ± 125	58 ± 290	0.7	0.453
Pitch contour percept.	932 ± 224	930 ± 232	1 ± 341	0.1	0.987
Pitch discrimination	864 ± 230	794 ± 175	69 ± 291	0.8	0.413
*Responsiveness*					
Pitch identification	51.7 ± 12.9	58.0 ± 1.9	−6.2 ± 13.8	−0.2^a^	0.785
Pitch contour percept.	48.0 ± 16.7	40.0 ± 17.9	8.0 ± 28.1	1.1	0.266
Pitch discrimination	44.9 ± 19.1	42.6 ± 20.9	2.2 ± 32.3	0.2	0.786
*Self-rated motivation*	7.9 ± 1.4	8.0 ± 1.5	−0.1 ± 1.9	−0.2^a^	0.859

*Accuracy* = correct responses. *Responsiveness* = correct plus incorrect responses. Degree of freedom = 22. ^a^ non-parametric Mann–Whitney U-test because of a ceiling effect. *p < 0.050.

**Figure 1:**
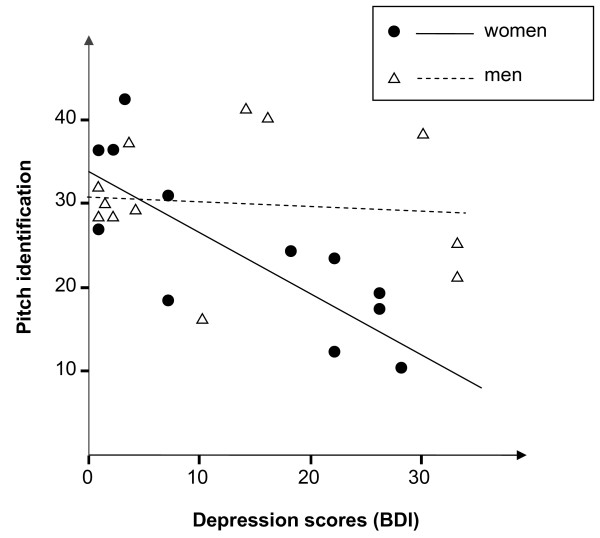
Linear regressions between depression scores (BDI) and number of correct responses in the pitch identification task differentiated by sex.

The analysis of further covariates revealed a sex effect. Female but not male depressive patients performed poor in pitch identification (female vs. male depressive patients: *Z* = −2.2, *p* = 0.025; female patients vs. female controls: -14.5 ± 9.5 with 45% fewer correct responses, one-tailed *Z* = −2.4, *p* = 0.031; male patients vs. male controls: -0.8 ± 10.6, one-tailed *Z* = −0.1, *p* > 0.500). Differential correlations of BDI depression scores with pitch identification reflect this sex effect: A high BDI score predicted inaccurate pitch identification only in women (*r*(12) = −0.82, *p* = 0.001; *β* = −0.73, *R²* = 0.68) but not in men (*r*(12) = −0.10, *p* = 0.745; *β* = −0.06, *R²* = 0.01; see Figure 1). Types of drugs did not differ between sexes (*χ²* = 0.4, *p* = .649). The difference in body weight failed significance (women: 70.6 ± 15.0 kg, men: 86.0 ± 14.0 kg, Z = 1.4, *p* = 0.180) thus an interaction between sex and relative dosage appears unlikely. Finally, impaired pitch identification was not significantly associated with age (*r*(12) = 0.28, *p* = 0.379), musical experience (*Z* = −0.6, *p* = 0.564), education (*χ²* = 5.4, *p* = 0.062 with a tendency to a lower effect in high-educated participants), or smoking (*Z* = 1.1, *p* = 0.299). Thus, depression and no other factor led to impaired pitch identification in women.

## Discussion

Pitch identification was selectively impaired in depression. Pitch identification differed from other pitch-related skills, and this supports a model of circumscribed cognitive deficits rather than a global impairment. The choice reaction task used for assessing pitch identification activated the intraparietal sulcus [11] and not memory-related mesio-temporal or frontal regions [23]. The intraparietal sulcus was associated with the encoding of numerosity [24]. Conceivably, numerosity contributed to poor performance of depressive patients during effortful tasks in the study of Hamar et al. [15]. These authors increased effort by applying a higher number of stimuli. In view of these findings, we suggest that poor pitch identification reflects a reduced mental representation of numerous alternatives in depression.

We could not confirm a bias from speed-accuracy tradeoff, responsiveness, and motivation. Though several studies reported a slowing in depression (e.g. [6]), choice reaction time remains a controversial issue: neither the present study nor Chase et al. [25] found a deceleration. Possibly, speed becomes less important in difficult tasks [26]. Malone and Hemsley [1] hypothesized that demand characteristics of the task enhance responsiveness, which was not supported in the current study. Depressive patients performed worse during the pitch identification task, which demanded a response in each trial. The total number of responses did not differ between depressive and non-depressive participants. A previous study did not find a generally reduced responsiveness as well because false positive responses and omissions counterbalanced each other [27]. Finally, the data did not support low motivation as origin of cognitive impairment [16] but a distinction of neuropsychological deficits from motivation in depression [28].

Inaccurate pitch identification emerged in women but not in men. This sex effect was unexpected. In view of the low number of participants, this finding is exploratory and needs a replication. Another study reported hearing impairment in male depressive patients [29] – though on a very basic sensory level and not directly comparable to the pitch identification task. The test which presented the lowest pitch differences in the present study – pitch discrimination – did not distinguish depressive and non-depressive participants. Therefore it is unconceivable that sex interacted with pitch identification due to different perception thresholds. Poor pitch identification in depressive women resembled the outcome after serotonergic medication in healthy (male) participants [17]. Serotonergic neurotransmission and specific auditory impairments in depression may be associated; in this regard, differences between women and men have not been thoroughly investigated so far.

## Conclusions

Auditory processing has been considered as marker of depression reflecting an underlying serotonergic dysfunction [30]. In the present study, depression in women impaired pitch identification; the effect was task-specific, not biased from reaction time and motivational factors, and persisted despite treatment. Thus, we suggest that inaccurate pitch identification merits attention as a potential marker for depression in women.

## Competing interests

The authors declare that they have no competing interests.

## Authors’ contributions

MS conceptualized the study, analyzed the data, and drafted the manuscript together with KM. EZ recruited healthy participants and performed the entire experiment. MG recruited the patients and provided substantial improvements of the manuscript. KM participated in the statistical analysis and drafted the manuscript. All authors read and approved the final manuscript.

## Pre-publication history

The pre-publication history for this paper can be accessed here:

http://www.biomedcentral.com/1471-244X/12/32/prepub
